# oxLDL Downregulates the Dendritic Cell Homing Factors CCR7 and CCL21

**DOI:** 10.1155/2012/320953

**Published:** 2012-04-30

**Authors:** Thomas Nickel, Susanne Pfeiler, Claudia Summo, Reinhard Kopp, Georgios Meimarakis, Zeljka Sicic, Marius Lambert, Korbinian Lackermair, Robert David, Andres Beiras-Fernandez, Ingo Kaczmarek, Michael Weis

**Affiliations:** ^1^Medizinische Klinik und Poliklinik I, Campus Grosshadern, Ludwig-Maximilians University, 81377 Munich, Germany; ^2^Institute of Clinical Chemistry, Campus Grosshadern, Ludwig-Maximilians University, 81377 Munich, Germany; ^3^Department of Surgery, Campus Grosshadern, Ludwig-Maximilians University, 81377 Munich, Germany; ^4^Department of Cardiothoracic Surgery, J. W. Goethe University, 61590 Frankfurt, Germany; ^5^Department of Cardiac Surgery, Campus Grosshadern, Ludwig-Maximilians University, 81377 Munich, Germany

## Abstract

*Introduction*. Dendritic cells (DCs) and oxLDL play an important role in the atherosclerotic process with DCs accumulating in the plaques during plaque progression. Our aim was to investigate the role of oxLDL in the modulation of the DC homing-receptor CCR7 and endothelial-ligand CCL21. *Methods and Results*. The expression of the DC homing-receptor CCR7 and its endothelial-ligand CCL21 was examined on atherosclerotic carotic plaques of 47 patients via qRT-PCR and immunofluorescence. *In vitro*, we studied the expression of CCR7 on DCs and CCL21 on human microvascular endothelial cells (HMECs) in response to oxLDL. CCL21- and CCR7-mRNA levels were significantly downregulated in atherosclerotic plaques versus non-atherosclerotic controls [90% for CCL21 and 81% for CCR7 (*P* < 0.01)]. *In vitro*, oxLDL reduced CCR7 mRNA levels on DCs by 30% and protein levels by 46%. Furthermore, mRNA expression of CCL21 was significantly reduced by 50% (*P* < 0.05) and protein expression by 24% in HMECs by oxLDL (*P* < 0.05). *Conclusions*. The accumulation of DCs in atherosclerotic plaques appears to be related to a downregulation of chemokines and their ligands, which are known to regulate DC migration. oxLDL induces an *in vitro* downregulation of CCR7 and CCL21, which may play a role in the reduction of DC migration from the plaques.

## 1. Introduction

Atherosclerosis is a dynamic inflammatory disease [1, 2] in which autoantigenes, such as oxidized low-density protein (oxLDL), play an essential role. Dendritic cells (DCs) are key regulatory antigen-presenting cells (APCs) that induce inflammatory processes. DCs can be detected in the vascular inflammatory environment, from endothelial dysfunction to consecutive plaque formation and rupture [3–5]. DCs are the most potent APCs and are highly specialized to prime naive T-cells [6]. Interestingly, mature DC-specific markers, for example, CD83, accumulate during plaque progression. It has been shown previously that the presence of CD83 is more than two fold, higher in symptomatic compared to asymptomatic patients [7].

DCs, after entering the vascular tissue, screen the environment for potential antibodies [2]. After processing the antigens, a maturation process is initiated and immunomodulatory receptors, such as the CD83-receptor (important for T-cell-stimulation), are upregulated [8]. In a previous study, we found a CD83 upregulation on DCs by proatherosclerotic stimuli like oxLDL and asymmetric dimethylarginine (ADMA) [9]. Beside CD83, DC induces CCR7 expression during maturation, which acts as a receptor for constitutively expressed CCL21 and CCL19 [10, 11]. In analogy to DC accumulation and maturation, activated CCR7 expressing T-cells are trapped especially in the plaque shoulders [12, 13] where mature DCs are forming clusters with T cells [13].

Several studies have demonstrated the important link between DC/T-cell recruitment and CCR7 expression [12–14]. The increase in CCR7 receptors induces homing of mature DCs and T-cells to the lymph nodes through CCL21/CCL19 expressing lymph vessels [2, 11, 12].

High plasma concentrations of oxLDL and their appearance in atherosclerotic lesions are of utmost importance in the pathogenesis of atherosclerosis [15].

The present study focuses on the influence of oxLDL on the DC-related chemokine receptors CCR7, CCL19, and CCL21. We characterized the DC-specific chemokine-ligand expression in human atherosclerotic carotid artery plaques. Furthermore, we investigated the impact of oxLDL on the DC receptor CCR7 expression and its ligands CCL-21 on human microvascular endothelial cells (HMECs).

## 2. Materials and Methods

### 2.1. *In Vivo*

#### 2.1.1. Study Population

 All investigations were approved by the institutional review board and the ethics committee of the Ludwig-Maximilians University of Munich. Informed consent was obtained from all patients. The investigation conforms to the principles outlined in the declaration of Helsinki. Between September 2006 and May 2008, carotid endarterectomy (CEA) was performed in 47 patients. The indications for CEA were based on carotid duplex sonography: stenosis of the internal carotid artery of more than 70% for symptomatic patients and more than 85% for asymptomatic patients. For comparison, we collected 10 blood samples from healthy age-matched men without clinically manifested atherosclerosis and 14 pieces of aortic tissue with no visible atherosclerotic lesions.

#### 2.1.2. RNA Isolation from Atherosclerotic Plaque for Real-Time PCR

 100 mg plaque tissue was homogenized in 1 mL QIAzol Lysis. 50 pg/tube RNA (PBMCs) was used. First-strand cDNA synthesis and PCR were performed using Omniscript from Qiagen (Hilden, Germany). The two-step quantitative real-time PCR (rt-PCR) system was applied according to the manufacturers' instructions. The quantitative real-time PCR system provides optimal performance with SYBR Green primers (Qiagen: Hilden, Germany). Rt-PCR was performed in the ABI PRISMTM 7700 System (Applied Biosystems, Germany). Data analysis was performed using the delta-delta-Ct method as described previously [16]. Primer sequences for the amplified fragments were GAPDH: 5′-CGG AGT CAA CGG ATT TGG TCG TAT-3′/5′-AGC CTT CTC CAT GGT GGT GAA GAC-3′; CCR7: 5′-TGG AGG CCT TTA TCA CCA TC-3′/5′-TGT AGG GCA GCT GGA AGA CT-3′; CCL19: 5′-CTG TGA CCC AGA AAC CCA TC-3′/5′-GCT TCA TCT TGG CTG AGG TC-3′; CCL21: 5′-CCC AGC TAT CCT GTT CTT GC-3′/5′-TCA GTC CTC TTG CAG CCT TT-3′; CD4: 5′-AGG AAG TGA ACC TGG TGG TG-3′/5′-CTC AGC AGA CAC TGC CAC AT-3′.

#### 2.1.3. Immunofluorescence Analysis of CCL21 Expression in Human Plaque and Aortic Tissue

 Frozen sections (10 *μ*m) were prepared from human aorta and plaque material of the internal carotid artery. After fixation in methanol/ethanolic acid for 1.5 min at −20°C, the sections were washed immediately 3 times for 3 min in PBS. Unspecific binding was blocked with a 2% BSA/0.2% Tween 20 solution for 1 h and followed by 3 washing steps in PBS. To visualize nucleoli we used DAPI (4′, 6′Diamidino-2′phenylindole, Merck KGaA, Germany) in a final concentration of 1 *μ*g/mL. After another washing step the sections were incubated with a fluorescent labelled anti-human CCL21/6Ckine antibody (5 *μ*g/mL, R&D Systems, Inc., Germany).

To determine the localization of the CCL21 expressing structure (CCL21/6Ckine antibody 5 *μ*g/mL, R&D Systems, Inc., Germany), the sections were also incubated with a Cy3-labeled anti-PECAM-1 antibody (5 *μ*g/mL, AbD Serotec, Germany), which demarcates the endothelia cells in the vessel wall. Immunofluorescence analysis of CCL21 expression was investigated by confocal microscopy (LSM 510 META, Zeiss, Plan-Neofluar 40x/1.30 oil objective, Germany) and quantified by LSM Image Browser 4.2 (Zeiss, Germany).

#### 2.1.4. Immunofluorescence Analysis of CCR-7/CD4- and CCR-7/CD83-Coexpression in Human Plaque and Aortic Tissue

 The sections were incubated with an FITC-labelled anti-human CCR7 antibody (1 : 100 concentration, R&D Systems, Inc., Germany) for 1 h, followed by 3 washing steps in PBS.

The sections were incubated with an Atto594-labeled anti-CD4 antibody (10 *μ*g/mL, AbD Serotec, Germany) or Atto594-labelled anti-CD83 (10 *μ*g/mL, AbD Serotec, Germany) for 1 h, which represents the T-lymphocytes (CD4) or mature DC (CD83). The immunofluorescence analysis was investigated by confocal microscopy (LSM 510 META, Zeiss, Plan-Neofluar 40x/1.30 oil objective, Germany).

#### 2.1.5. ELISA for oxLDL and CCL21

 Plasma tubes were centrifuged and the plasma was fractionated and frozen at −80°C.

*oxLDL levels* were examined by using a cytokine-specific ELISA kit according to the manufacturers' instructions (ImmunDiagnostik, Bensheim, Germany) [17]. Before analysis, samples were treated as described above. In brief, after washing with wash buffer several times, standard samples and controls were added and incubated for 4 hours at room temperature on a horizontal mixer. Thereafter, a washing conjugate was added and incubated for 1 h as described before. The substrate was added and incubated for 25 minutes in the dark. Stop-solution was added and immediately analyzed by an ELISA-reader at 450 nm.

Detection of *CCL21 in serum* was also performed by a quantitative sandwich enzyme immunoassay technique, according to the manufacturers' instructions (R&D Systems, Germany).

### 2.2. *In Vitro*

#### 2.2.1. Generation of Monocyte-Derived Dendritic Cells (DCs)

 Mononuclear cells were isolated from 100 mL of peripheral blood of a healthy human donor by a Ficoll density gradient according to the protocol by Boyum [18]. The purity of the monocyte culture was enhanced up to 97% by adhesion on *γ*-globulin coated plates. DCs were obtained from the monocyte culture according to the modified protocol by Romani et al. [19] as described in detail in our recent publication [9, 17].

#### 2.2.2. Endothelial Cells (ECs)

 All experiments were performed with human microvascular endothelial cells (HMECs-1), generously provided by the Center for Disease Control and Prevention and the National Center for Infectious Disease (Atlanta, USA). Cells were cultured in MCDB-131 (without phenol red; cc pro, Neustadt/W., Germany) supplemented with 10% fetal calf serum (FCS; PAA, Pasching, Austria), 2 mM L-Glutamine (Biochrom AG, Berlin, Germany), 1 *μ*g/mL hydrocortisone, and 10 ng/mL epidermal growth factor (Sigma, Taufkirchen, Germany). The cells were used at least 10 days after thawing and for no more than 20 passages [20].

#### 2.2.3. LDL Oxidation

 LDL (density = 1.019 to 1.063 g/mL) was isolated from human plasma of normolipidemic healthy volunteers by sequential ultracentrifugation as described and stored in PBS containing 2 mmol/L EDTA. Shortly before oxidation, the EDTA was removed from LDL by passing the lipoprotein through a PD 10 column (Pharmacia, Austria). LDL was oxidized in Ham's F-10 medium by exposure to 5 *μ*mol/L CuSO_4_ at 37°C for 30 h [11, 21]. All preparations were dialyzed before they were added to the cultured cells. This reflects oxLDL concentrations in the plaque and has been used in previous studies [5, 22, 23]. In addition, several other studies have shown that an oxLDL concentration of (10 *μ*g/mL) is not toxic to the cultured cells [9, 24, 25]

#### 2.2.4. Total RNA Isolation and Real-Time PCR

 For isolation of mRNA from HMECs-1 and DCs, the total RNA isolation RNAeasy Mini Kit from Qiagen (Hilden, Germany) was used according to the instructions provided by the manufacturer. 50 pg/tube RNA (PBMC) was used. First-strand cDNA synthesis and PCR were performed using Omniscript from Qiagen (Hilden, Germany). The two-step quantitative RT-PCR system was applied according to the manufacturers' instructions and as described above [16].

#### 2.2.5. Flow-Cytometric Analysis of DCs

 Cells were incubated with antibodies according to the manufacturers' instructions. Fixed cells were analyzed on an FACS Calibur cytometer (Becton Dickinson). Antibodies were matched to iso-type-controls (Mouse-*γ*2a-(FITC)/-*γ*1(PE)-Fastimmune; BD; USA). To verify the purity of our DC-culture, we used CD3 (BD, USA) and CD20 (BD, USA) to rule out a T-cell and B-cell contamination (<5%). DCs were characterized by low CD14 expression (BD, USA) and high expression of CD80 (BD, USA), CD86 (BD, USA), and HLA-DR (BD, USA), as described previously [26].

At day five, we incubated the DCs with oxLDL (10 *μ*g/mL) for 24 hours. Expression analysis of the chemokine receptor CCR7 (R&D, USA) and CD83 (BioLegend, USA) on human DCs were performed using flow-cytometer analysis [16, 17].

#### 2.2.6. Immunofluorescence of CCL21 on HMECs-1

 HMECs-1 were cultivated on chamber slides and stimulated with 10 *μ*g/mL oxLDL for 48 h. The monoclonal antibody for CCL21/6Ckine (R&D, USA) was labeled with Alexa 488 using the Alexa Fluor Protein Labeling Kit (Invitrogen, life technologies, USA) according to the manufacturers' instructions. HMECs-1 were incubated with the labeled antibody (concentration 1 : 20) for 30 minutes. Thereafter, Vybrant DiD (Invitrogen, life technologies, USA) was used as a marker of the plasma membrane. CCL21-labeled HMECs-1 were investigated by confocal microscopy (Zeiss LSM 510 Meta, Plan-Apochromat 63x/1.40 oil objective) and quantified by LSM Image Browser 4.2 (Zeiss, Germany).

#### 2.2.7. Statistical Analysis

 Data is presented as ± standard-deviation of the mean (SDM). The Kolmogorov-Smirnov test was used to determine whether or not the data was normally distributed. If the data was normally distributed, the unpaired *t*-test was used to compare the two groups. Data that was not normally distributed was compared using the Wilcoxon signed Rank Test. Differences between means were considered significant with *P* < 0.05 and highly significant with *P* < 0.01. All *in-vitro* experiments were repeated at least eight times with different cells and lipoprotein preparations. SPSS (version 16, Leibniz Rechenzentrum Munich) was used for statistical analysis.

## 3. Results and Discussion

### 3.1. *In Vivo*

#### 3.1.1. Study Population

*Carotid plaque tissue and blood samples* were collected from 47 patients undergoing CEA, of which 77% were male and, on average,  70 ± 8  years of age. 66% had asymptomatic disease, whereas and the rest showed cerebral ischemic complications. The different risk profiles and medication are summarized in Table 1.

*Serum for the PBMCs in the control group *was collected from 10 healthy age-matched patients. 60% were of male gender with an average age of 68 ± 4 years. None had cerebral ischemic complications or clinical manifest atherosclerosis. Concerning the risk-profile, 20% had insulin-dependent diabetes mellitus, 80% hypertension, and 10% were smokers. Concerning the medication, 10% took thrombocyte aggregation inhibitors, 80% statins, and 60% had a *β*-blocker (Table 1).

*Aortic tissue in the control group* was collected from 14 patients who underwent valve replacement and had no sign of aortic atherosclerosis. 71% were of male gender with an average age of  64 ± 5  years. 14% had a cerebral ischemic event. Concerning the risk profile, 28% had insulin-dependent diabetes mellitus, 85% hypertension, and 35% were smokers. With respect to medication, 43% took thrombocyte aggregation inhibitors, 78% statins, and 43% had a *β*-blocker.

#### 3.1.2. CCL21/CCL19 and CD83/CCR7 and CD4 mRNA Analysis of the Atherosclerotic Plaque

In line with previous investigations, we detected that the median CD83 mRNA levels in plaque tissue from CEA-group were nearly twice as high as in healthy aortic tissue (*P* < 0.01), demonstrating the augmented presence of mature DCs in the atherosclerotic tissue [7, 27, 28]. In a subgroup analysis, we further found that CD83 levels in men were significantly higher (+120%; *P* < 0.05) compared to women. CD4 mRNA levels were even  23 ± 11  fold higher in healthy aortic tissue (*P* < 0.01; data not shown).

CCR7, normally coexpressed on mature DCs and T-cells, was 81% lower in the plaque compared to compared to healthy aortic tissue (*P* < 0.01) without any gender specific differences.

**Figure 1:**
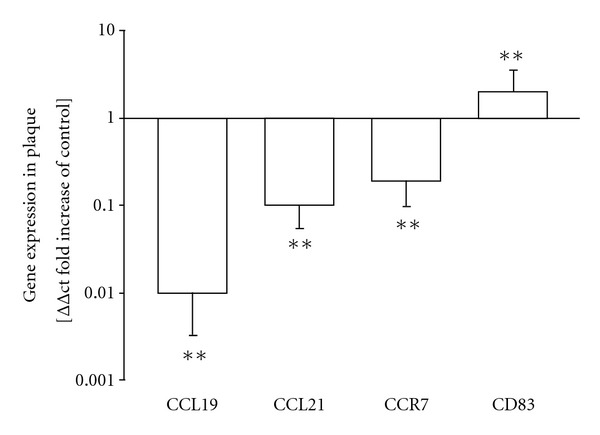
mRNA expression of CCL21/19, CCR7, and CD83 in vascular plaques compared to aortic tissue without plaque detection. *In vivo* changes (relative fold increases) in mRNA expression of CCL19, CCL21, CCR7, and CD83 in carotid plaques compared to healthy aortic material. *P < 0.05, **P < 0.01, (*n* = 47) versus aortic tissue (*n* = 14).

Further analysis of CCL19 revealed that its transcripts were downregulated by 99% (*P* < 0.01). CCL21 was found 90% lower in the plaque (*P* < 0.01) compared to healthy aortic tissue (Figure 1).

Furthermore CCL21 was also found lower in the symptomatic patients (58%; *P* < 0.05) compared to asymptomatic patients from the CEA group.

#### 3.1.3. Immunofluorescence Analysis of CCL21 Expression in Human Plaque and Aortic Tissue

 To confirm the PCR results on the protein level, we performed a semiquantitative immunofluorescence analysis on histological slides of human aortic and plaque tissue.

**Figure 2:**
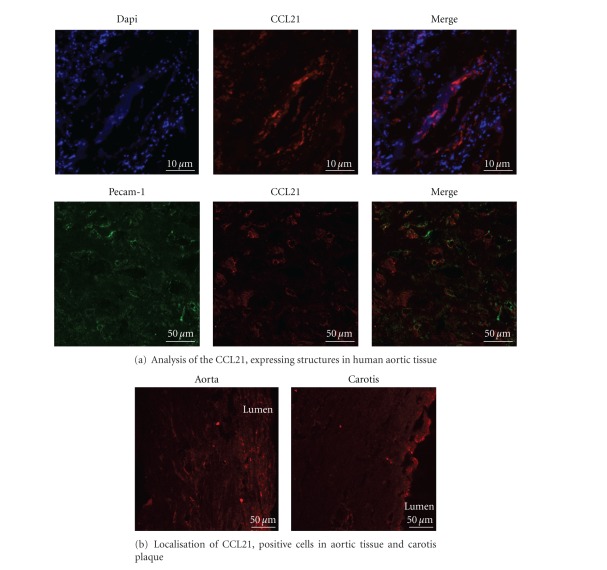

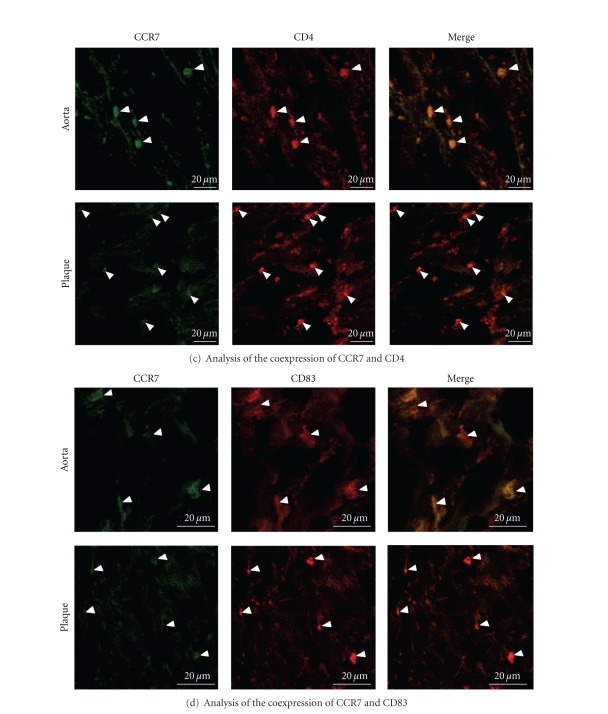
Protein expression of CCL21 and CCR7 on carotid plaque and aortic tissue. (a) Analysis of the CCL21 expressing structures in human aortic tissue. In the upper part the blue staining represents the cell nuclei (DAPI) and the red staining represents CCL21, positive structures (*α*-CCL21/6Ckine-Alexa488, pseudocolored). Scale bar 10 *μ*m. To examine the vessel wall as CCL21 expressing structures (lower part) in human aorta tissue endothelial cells were stained with a PECAM-1 antibody (green, *α*-PECAM-1-Cy3, pseudocolored). The red staining represents CCL21 (*α*-CCL21/6Ckine-Atto594). The merged picture shows a co-localization of CCL21 and PECAM-1 in the vessel wall of the human aorta. Scale bar 50 *μ*m. (b) The representative pictures show differences in the expression level of CCL21 (red, *α*-CCL21/6Ckine-Atto594) in aorta and carotid plaque tissue. A decreased CCL21-protein expression in carotid plaque compared to aorta tissue was shown by immunofluorescence microscopy. CCL21 is primarily expressed by the vasa vasorum of the vessel. Scale bar 50 *μ*m. (c) A decreased CCR7 expression (green, *α*-CCR7-FITC) on CD4 positive cells (red, *α*-CD4-Atto594) was detectable in plaque tissue compared to aorta tissue. Arrows indicate the cells. Scale bar 20 *μ*m. (d) Also a decreased CCR7 expression (green, *α*-CCR7-FITC) on CD83, positive cells (red, *α*-CD83-Atto5994) in aorta and plaque tissue is shown in the representative pictures. Arrows indicate the cells. Scale bar 20 *μ*m.

To analyze the location of CCL21 expressing cells, we stained the endothelial cells (*α*-PECAM-1-Cy3) in the aortic tissue section. This revealed a colocalization of the CCL21 and the vascular wall of the vasa vasorum (Figure 2(a)).

In every single healthy aortic tissue slide (*n* = 6), we could detect higher CCL21 expression via an increased fluorescence signal signal from the binding fluorescent antibody than in the plaque tissue (*n* = 15). An example is shown (*n* = 15; in Figure 2(b)).

#### 3.1.4. Immunofluorescence Analysis of CCR-7/CD4 and CCR-7/CD83 Coexpression in Human Plaque and Aortic Tissue

To further investigate differences of the CCR7 level on T-lymphocytes (CD4 positive) and mature DC (CD83 positive) in human tissue, immunofluorescence analysis was used. By double staining, we could detect a decreased signal of CCR7 on CD4-positive cells within the plaque tissue (Figure 2(c); lower panel) compared with control aortic tissue (Figure 2(c); upper panel). Also, CD83-expressing cells within the plaque show a decreased CCR7 signal (Figure 2(d); lower panel) compared to the aortic tissue which correlates with the PCR results.

#### 3.1.5. Circulating oxLDL and CCL21 Concentrations

For oxLDL, we found a 52.1% (*P* < 0.05) higher concentration in the plaque group compared to the control group (Table 1). oxLDL serum concentrations showed no correlation with CCL21/CCL19 or CCR7 expressions in plaques (Table 1).

For CCL21, we found a 28% (*P* < 0.01) higher serum concentration in the CEA group compared to the controls (Table 1). 

### 3.2. *In Vitro* Cell Experiments

#### 3.2.1. CCR7 and CCL21/CCL19 Expression in DCs and HMECs-1

**Figure 3:**
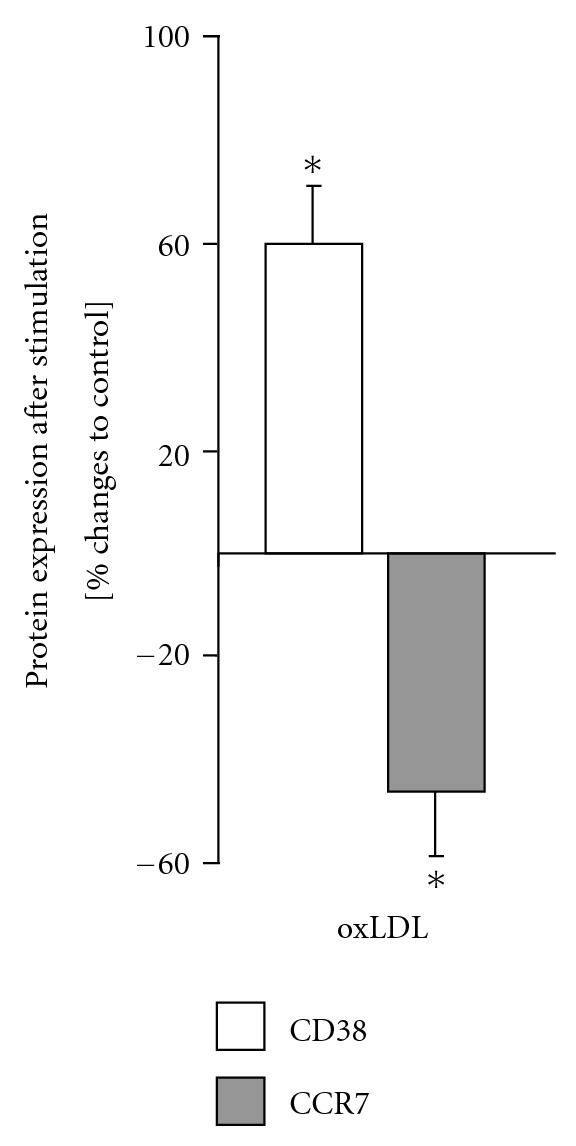
Protein expression of CD83 and CCR7 on DCs after stimulation with oxLDL. An increase of the expression of CD83 and a decrease of the expression of CCR7on DCs after stimulation with oxLDL (10 *μ*g/mL) was observed. Positive cells were measured by flow cytometry and the percent (%) changes were plotted in the figure. *P < 0.05, (*n* = 8) versus unstimulated controls.

After stimulation with oxLDL, we found a reduction of the mRNA levels of CCR7 (DCs) by 30% using RT-PCR (*n* = 8; *P* < 0.05). This correlated with a reduction in protein expression by 46% (*n* = 8; 25.7 ± 1.06% versus control 47.6 ± 19.3% positive cells; *P* < 0.05; Figure 3). In contrast, protein expression of CD83 was significantly upregulated by 10 *μ*g/mL oxLDL, as shown in Figure 3 (60%, 46.9 ± 11.3 versus control 29.3 ± 17.7% positive cells; *P* < 0.05; Figure 2) by 10 *μ*g/mL oxLDL (Figure 3).

**Figure 4:**
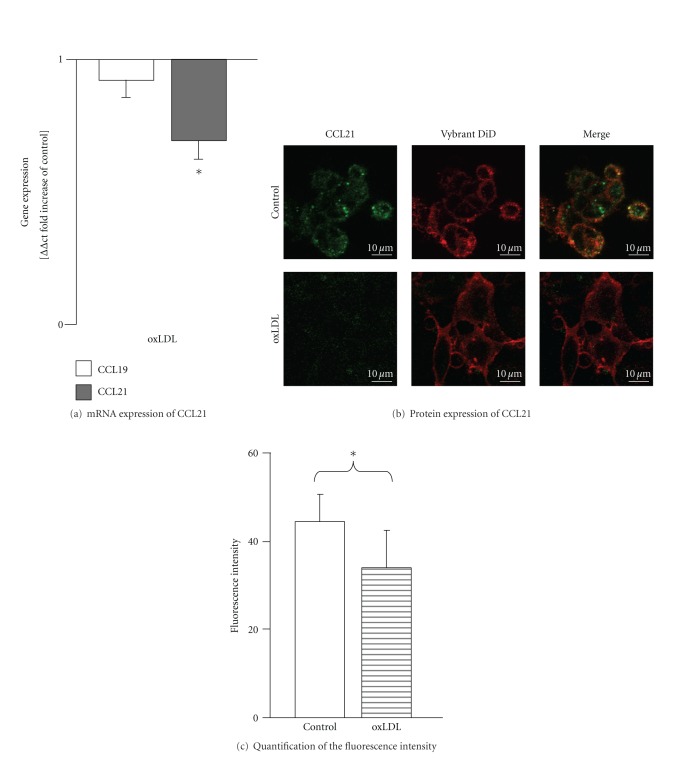
mRNA expression of CCL21/19 and protein expression of CCL21 on HMECs-1 after stimulation with oxLDL. (a) Fold increase in mRNA expression levels of CCL21/19 in human microvascular endothelial cells after 48 h of stimulation with oxLDL (10 *μ*g/mL) analyzed by real-time PCR. *P < 0.05, (*n* = 9) versus unstimulated controls. (b) Corresponding protein expression of CCL21 measured by immunofluorescence analysis. The green staining represents CCL21 positive structures (*α*-CCL21/6Ckine-Alexa488), red represents the plasma membrane (Vybrand DiD). Scale bar 10 *μ*m. (c) The fluorescence intensity was quantified showing a significant change for oxLDL. *P < 0.05, (*n* = 8) versus unstimulated control.

For HMECs-1, stimulation with oxLDL led to a significant reduction of CCL21 mRNA expression (50%; ΔΔct = 0.5; *P* < 0.05, Figure 4(a)). These results correspond with the results of the immunofluorescence analysis, where we found a 24% down-regulation of CCL21 receptor expression (*n* = 9; 34.00 ± 8.53 versus control 44.54 ± 6.23 intensity/mm^2^; *P* < 0.05; Figures 4(b) and 4(c). oxLDL had no significant impact on protein or mRNA expression of CCL19 (Figure 4(a)).

## 4. Discussion

The main findings obtained from our study are (1) The DC-chemokine receptor CCR7 and its ligands CCL21/CCL19 are significantly downregulated in atherosclerotic plaques; (2) Circulating CCL21 levels are upregulated in serum from atherosclerotic patients; (3) oxLDL impairs CCR7 expression in DCs and CCL21 expression in microvascular ECs.

Our data support the concept that modulation of chemokine receptors (mediated, e.g., by oxLDL) in the plaque may trigger retention of DCs, thereby impeding the vascular innate and adaptive immunity [29, 30]. Accordingly, Angeli et al. found that oxLDL and other lipid mediators are jointly responsible for trapping of DCs in the vascular wall [29]. In our *in vivo* study, oxLDL serum concentrations showed no correlation with CCL21/CCL19 or CCR7 expression in plaques. However, CCR7 and CCL21/CCL19 were downregulated in atherosclerotic plaques compared to nonatherosclerotic aortic tissue. This discrepancy may be explained by the fact that oxLDL is unstable in serum but accumulates in the subintimal space over time. Hereby, it reaches much 70 fold higher subintimal concentrations as compared to serum levels [31]. This fact especially appeases to monocytes rich plaques [31].

We found CCR7 expression to be downregulated after 24 hours of stimulation with oxLDL. Alongside, CCL21 was downregulated on HMECs-1 after incubation with oxLDL, in a concentration which predominates in atherosclerotic plaques. Trogan et al. previously demonstrated that phases of hyperlipidemia severely reduce CCR7 mRNA and the corresponding protein levels in an ApoE^−/−^-mice model. CCR7 increased in the presence of normolipidemia. In their work, CCR7 expression was found to be essential for the migration of DCs from atherosclerotic plaque. In atherosclerotic plaques of ApoE^−/−^-mice, CCR7 receptor expression was reduced. When transferred to wild-type mice, this process was accompanied by an upregulation of CCR7 itself [32].

Under a high-dose statin therapy, Damas et al. found a significant decrease in CCL19 and CCL21 levels in serum [2]. Our data support this observation, showing reduced CCL21 serum protein expression in the control group, characterized by low oxLDL levels. The difference in controls was evident despite a low-dose statin therapy.

Our results suggest that DC maturation is triggered by increased oxLDL deposits in the subintimal space. At the same time DCs, and even T-cells do not seem to express enough CCR7 receptors in order to migrate. As a consequence, oxLDL, DCs and T-cells appear to accumulate, thereby enhancing plaque inflammation processes and possibly plaque rupture.

These results complement the former findings that oxLDL increased the adhesion and promotes the maturation and differentiation of DCs from monocytes [9, 26]. Furthermore, oxLDL supports the building of foam-cell accumulations and also directly induces chemotaxis of immune cells like T-cells via upregulation of endothelial adhesion molecules [9]. Keeping in mind that DCs are responsible for priming naive T-cells to oxLDL-specific T-cells [9, 33], the interaction of oxLDL and DCs must be taken into account as a key factor for the induction and progression of atherosclerosis.

oxLDL not only seems to have an influence on the expression of CCR7, but also to induce downregulation of CCL21 on ECs *in vitro* as well as decreased expression of CCL21 and CCL19 in the plaque. Comparing CCL19 with CCL21, the latter appears to be affected more directly by local plaque homing factors. In plaque versus normal aortic tissue, we found a much higher response to CCL21 on ECs as opposed to CCL19. Typically, the expression of CCL21 on ECs is higher than CCL19 [11], since CCL21 has a C-terminus indicating a strong affinity to glycosaminoglycan. This is crucial for effective presentation of CCL21 on ECs [11, 34].

In contrast to our study, other investigators demonstrated an upregulation of CCL21/CCL19 in carotid plaques [7]. In previous studies, 2–4 coronary or renal vessels from autopsy material were taken as controls. To overcome these limitations, we used 14 age-matched healthy aortic tissue samples taken from valve operations serving as controls, which were immediately frozen after their operative preparation, similar to the procedures of carotid tissue preparation. Autolysis and coincidence caused by the small number of controls were therefore ruled out in our experimental setting. A further aspect which points against the renal vessels as controls is the fact, that the vascular-associated lymphoid tissue (including DC and T-cells) has been characterized in human large arteries, including the aorta, carotid, and coronary arteries [35, 36] but not in renal vessels.

## 5. Conclusions

In summary, our results suggest that alterations in the vascular chemokine profile may be responsible for accumulation of mature DCs in plaque, which potentially enhance the risk for plaque rupture. It may be argued that inhibiting DC migration leads to a less robust immune response. Indeed, altered priming can be expected in the presence of impaired DC migration. However, if migration is blocked and maturation enabled, the consequence of trapping mature DCs in the direct atherogenic vicinity may induce plaque formation and progression or even rupture. Suppression of DC migration and maturation may, therefore, prove a promising future therapeutic target to prevent plaque instability and rupture.

## Figures and Tables

**Table 1:** Clinical characteristics of the patients and controls. Clinical characteristics including age, sex, cardiovascular risk factors, relevant medication, oxLDL and CCL21 of patients who received CEA and healthy controls without clinical history of manifest atherosclerosis.

	CEA-patients	healthy controls
	(*n* = 47)	(*n* = 10)
Age (years)	70 ± 8	68 ± 4
men % (*n*)	77 (36)	60 (6)
TIA/CI % (*n*)	32 (15)	0 (0)
ID DM % (*n*)	26 (12)	20 (2)
Hypertension % (*n*)	91.5 (43)	80 (8)
Hyperlipidemia % (*n*)	85 (40)	80 (8)
Nicotine abusus % (*n*)	38 (18)	10 (1)
ASS/Clopidogrel % (*n*)	91.5 (43)	10 (1)
*β*-blocker % (*n*)	60 (28)	60 (6)
Statins % (*n*)	79 (37)	80 (8)
oxLDL (ng/mL)	207.3 ± 240.2	99.4 ± 20.3
CCL21 (pg/mL)	1335.1 ± 771.5	961.2 ± 210.9
